# High‐resolution photo‐mosaic time‐series imagery for monitoring human use of an artificial reef

**DOI:** 10.1002/ece3.2342

**Published:** 2016-09-09

**Authors:** Georgina Wood, Tim P. Lynch, Carlie Devine, Krystle Keller, Will Figueira

**Affiliations:** ^1^ School of Biological Sciences Coastal & Marine Ecosystems Group University of Sydney Sydney New South Wales 2000 Australia; ^2^ CSIRO Oceans and Atmosphere – Coasts GPO Box 1538 Hobart Tasmania 7001 Australia; ^3^ CSIRO National Research and Collections Australia GPO Box 1538 Hobart Tasmania 7001 Australia; ^4^ School of Biological, Earth and Environmental Sciences Evolution & Ecology Research Centre UNSW Sydney New South Wales 2052 Australia

**Keywords:** bait fishery, coastal use, CRAGS, fishing effort, Gigapan, high‐resolution photo‐mosaic, monitoring, party size, pelagic fishery, remote camera

## Abstract

Successful marine management relies on understanding patterns of human use. However, obtaining data can be difficult and expensive given the widespread and variable nature of activities conducted. Remote camera systems are increasingly used to overcome cost limitations of conventional labour‐intensive methods. Still, most systems face trade‐offs between the spatial extent and resolution over which data are obtained, limiting their application. We trialed a novel methodology, CSIRO Ruggedized Autonomous Gigapixel System (CRAGS), for time series of high‐resolution photo‐mosaic (HRPM) imagery to estimate fine‐scale metrics of human activity at an artificial reef located 1.3 km from shore. We compared estimates obtained using the novel system to those produced with a web camera that concurrently monitored the site. We evaluated the effect of day type (weekday/weekend) and time of day on each of the systems and compared to estimates obtained from binocular observations. In general, both systems delivered similar estimates for the number of boats observed and to those obtained by binocular counts; these results were also unaffected by the type of day (weekend vs. weekday). CRAGS was able to determine additional information about the user type and party size that was not possible with the lower resolution webcam system. However, there was an effect of time of day as CRAGS suffered from poor image quality in early morning conditions as a result of fixed camera settings. Our field study provides proof of concept of use of this new cost‐effective monitoring tool for the remote collection of high‐resolution large‐extent data on patterns of human use at high temporal frequency.

## Introduction

Artificial reefs are a global phenomenon, often deployed to enhance fishing or other activities such as SCUBA diving (Baine 2001; Jensen 2002; Sutton and Bushnell 2007). Deployment of artificial reefs can have negative consequences however, such as displacement of commercial fishing effort into the recreational sector (Sutton and Bushnell 2007), user conflict (Samples 1989) and stunting of ecological succession or collapse from overfishing (Jackson et al. 2001). There is also a long‐running debate over whether artificial reefs merely attract fish from other areas or actually contribute to production (Bohnsack 1989; Pickering and Whitmarsh 1997; Brickhill et al. 2005). A key metric to understand these issues is fishery effort; however, assessments are often constrained by costs. Traditional roving, access point or vantage point surveys via direct observation from platforms, cars, boats or air (Pollock et al. 1994) are, per replicate, expensive to collect, especially when reefs are only accessible by boat. A cheaper alternative is field deployment of remote cameras to monitor activity (Wise and Fletcher 2013). Cameras allow high replication over extended time periods, although there is often a compromise between the quality of images and size of the field of view (Allen et al. 1984; Brown and Litvaitis 1995). In the case of artificial reefs, often the distance from shore to the point where effort must be observed can limit the information that can be extracted from these standard systems. As a result, deployments for fisheries applications often place digital cameras or webcams at specific vantage or choke points and combine with in situ observations to validate user counts (Parnell et al. 2010; Smallwood et al. 2012; Hartill 2015) to estimate effort on target reefs indirectly. However, where access points and/or focal areas for fishing are numerous, onsite interviews are typically required to determine effort at specific areas and as such can be cost prohibitive.

This study describes the novel application of a ruggedized high‐resolution photomosaic (HRPM) camera system to directly estimate human use of an offshore artificial reef. HRPM systems have been used to capture very high‐resolution images at macro to medium spatial extents (<0.1 to 6 km; Nichols et al. 2009; Brown et al. 2012; Lynch et al. 2015). The CSIRO modified one of these HRPM systems to produce CSIRO Ruggedized Autonomous Gigapixel System (CRAGS), which can be deployed remotely in harsh environmental conditions to collect time series of gigapixel digital images (Hughes et al. 2014). In this trial, the CRAGS was deployed on the exterior railing of a lighthouse, which overlooks an artificial reef offshore from Sydney heads in NSW, Australia. Specific aims of the work were to (1) quantify boating activity and determine differences in use within and across days; (2) compare data from CRAGS to a webcam that was simultaneously monitoring the artificial reef; (3) determine whether high‐resolution panoramas could be used to distinguish more specific metrics of effort (activity type and party size); and (4) describe the costs, benefits, and limitations of both techniques.

## Methods

Human Ethics Permission was granted for this work by the Sydney University Human Research Ethics Committee, Project Number: 2014/928.

### Study site

**Figure 1:**
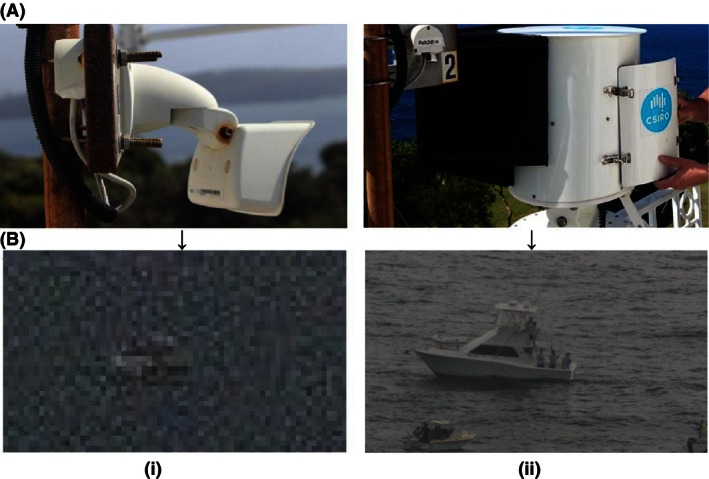
Photograph showing (A) i) webcam; and ii) CRAGS high‐resolution photo‐mosaic (HRPM) system with (B) comparative difference in i) webcam; and ii) HRPM image resolution.

The Sydney Offshore Artificial Reef (OAR) is located at 38 m depth, approximately 1.9 km south‐east of Sydney's South Head, NSW, Australia (33° 50.797′ S, 151° 17.988′ E) and is designed to enhance recreational fishing opportunities (NSW DPI, 2011). Daytime (06:00–18:00) use of the OAR for the period 26th January–14th February 2015 was estimated independently using two shore‐based photographic time series produced by CRAGS and a webcam. The cameras were installed side‐by‐side on a balcony railing 85 m above sea level at the South Head Old Signal Station 1.3 km from the OAR (33°51′1.47″ S, 151°17′12.41″ E, Fig. 1A). Prior to the study's initiation, the cameras’ field of view was set using a *Garmin* ^*™*^ global positioning system (GPS) by driving a boat to four points that marked the 200 m boundary on all sides of the OAR (total area 4 ha). The images from both cameras were visually analyzed for human activity in Windows photo viewer with a screen overlay indicating the focal area. Due to technical issues with one camera system, the 5th and 13th February were omitted from analysis.

### Remote camera systems

High‐resolution panoramas were produced by CRAGS, a modified GigaPan® EpicPro robotic HRPM system: GigaPan Systems, Portland, OR. This was modified with a new “thrall unit” chipset and software to program the robot via a USB interface. The new chipset radically reduced power requirements. Individual photographs were captured with a Cannon 600D EOS digital SLR camera Canon Inc, Chichibu‐shi, Saitama, Japan, through a Cannon EFS 70–300 mm lens with a Promaster Spectrum 7 2× Digital Teleconverter and a Kenko 58 mm digital UV filter onto a 128 GB SanDisk Extreme card. The system was encased in a custom‐built powder‐coated aluminum housing, with a shatter‐resistant glass window and 10‐kg *Hollyhook* ^*™*^ external battery pack, allowing for remote, long‐term deployments in harsh conditions, without the need of a battery changeover (Hughes et al. 2014).

CRAGS images were compared to images captured by a 3 megapixel M24M *Mobotix* ^*™*^ (8 mm focal length, 2.0 aperture) web camera with inbuilt housing and 32 GB mini SanDisk card. The CRAGS was set up in manual mode (zoom 250 mm, 1/125 shutter speed, F11, ISO 200) with focus set to the approximate location of the OAR and programmed to take one panorama of 135 images (image size 5184 × 3456; nine rows by 15 columns) every 30 min. Images were downloaded every 7 days (~1 h) with a total of 69,360 images collected. These were stitched to form 475 panoramas using the Gigapan Import wizard in Autopano Giga (Kolor, 64 Bit). Lighting balance was adjusted with the Colour Level Gamma tool before batch rendering to TIFF format.

### OAR activity monitoring

Vessels and party size were scored in the following categories: (1) Private fishing; (2) Charter fishing; (3) Spearfishing; (4) Commercial Fishing; (5) SCUBA Diving; and (6) Other (whale‐watching & motorboats). To ensure that the data did not include vessel transit, boats were only recorded if they appeared to be stationary in each photograph (i.e., no presence of a wake). If a boat was observed across >1 consecutive sample periods, it was noted as the same “trip”.

The webcam was set to automatic mode and captured a time‐stamped image (image size 2048 × 1536) of the study area every 60 sec. Images were downloaded fortnightly (~2 h download time) with a total of 14,742 images captured. The low resolution of the images meant that exact activities could not be determined (Fig. 1B), so any vessel present and remaining within the boundary for ≥5 consecutive images (i.e., ≥5 min) were considered to be using the OAR (Keller et al. 2016). Once a user vessel was established, time and duration of boating trips were recorded (~1‐day analysis for 1 week of data).

Previous work (Keller et al. 2016;.) suggested that webcam‐based counts underestimated actual counts compared with binocular counts. Therefore, an additional validated webcam estimate was generated so that the data from the two camera systems could be compared to in situ observations of boats. The validated estimate was calculated using a correction factor based on binocular observations made by marine rescue volunteers at the South Head Signal Station (Keller et al. 2016) over 57 days between November 2012 and February 2015 when the webcam was operating. The correction factor (*Cf;* Blumenfeld 2001) was calculated using the inverse of the slope from the regression equation:

Y=bx,

where *Y* = daily fishing events from webcam images; *x* = daily fishing events observed with binoculars.

$$Cf=\frac{1}{b}.$$

The *Cf* was multiplied by the daily webcam boat count estimates for the period of this study to generate a corrected webcam boat count estimate to which the CRAGS counts could be compared.

### Data analyses

To compare boat counts at the highest temporal resolution common to both methods, the number of boat trips captured by the webcam during each 8‐min period when the CRAGS was shooting was determined. The effect of time of day and method (CRAGS/webcam) on boat counts was tested using hierarchical log‐linear analysis (Sokal and Rohlf 1995) in SPSS v20 (IBM Corp, New York, NY). Counts were binned into dawn (06:00 and 06:30), morning (10:00 and 10:30), afternoon (14:00 and 14:30), and dusk (17:00 and 17:30). There was an effect of time of day due to differences in the dawn period (see Results). As there are no post hoc tests for log‐linear analyses, the effect of this dawn period on differences between methods was assessed by running a second hierarchical log‐linear analysis, which excluded the data collected during dawn.

As most use surveys generate daily estimates of activity levels, the total boating hours per day were also compared between the two methods. Total boating hours (*H*) per day were calculated as:

H=b×s

*b *= Σ *boat counts* for all images on a day; *s = sample interval* (*minutes*)/60.

Daily boating hours were then compared. Due to nonindependence between sample subjects (boats simultaneously measured by two methods), this was done with a two factor repeated‐measures ANOVA including day type (weekday or weekend) and method (CRAGS or webcam, the repeated‐measures factor) in Statistica (Statsoft Inc., Tulsa, OK, version 12, 2013). Because CRAGS samples were affected by low light at dawn (see Results), the model was run using estimates excluding samples collected before 07:00. Data was square root transformed to conform to the assumptions of normality and variance homogeneity (Quinn and Keough 2002).

Using the CRAGS estimates for hours per vessel spent at the OAR on weekdays and weekends, the relative proportion of different activities (private fishing, charter fishing, spearfishing, commercial fishing, SCUBA diving, or other) conducted on weekdays and weekends were estimated. Mean party size was calculated by counting the number of people on each boat at each sample.

## Results

In total over the 18‐day period, 147 boating trips were recorded by the CRAGS system and 169 by the webcam. These were less than the 183 trips predicted by the validated webcam data; 30 of the 475 CRAGS panoramas were unable to be scored due to inadequate lighting with 28 completely black images taken at 06:00 and 06:30 periods.

### Comparison of counts from each interval

**Figure 2:**
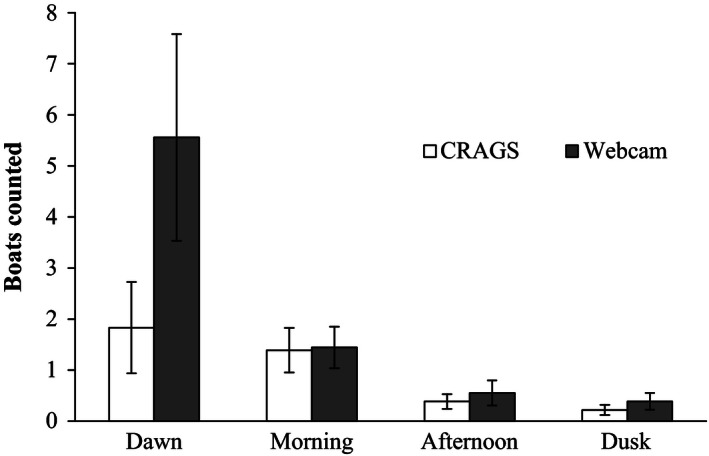
Average number of boats captured in images taken at dawn (06:00 and 06:30), morning (10:00 and 10:30), afternoon (14:00 and 14:30), and dusk (17:00 and 17:30) with the CRAGS high‐resolution photo‐mosaic system and webcam (± SE;* n* = 18).

Inclusion of data taken during 06:00 and 06:30 periods led to an interaction between time of day and method on the number of boats counted (Hierarchical log‐linear analysis: time*method: *χ* ^2^ = 10.36, 3 df, *P* < 0.05). However, this interaction did not occur when these data sets were excluded (time*method: *χ* ^2^ = 0.76, 2 df, *P* > 0.05). With the dawn data excluded, there was a difference in the number of boats across the day with boats recorded most frequently in the early morning and then decreasing throughout the day (Fig. 2; time: *χ* ^2^ = 33.3, 3 df, *P* < 0.05) but with no difference between methods (method: *χ* ^2^ = 0.76, 1 df, *P* > 0.05).

### Comparison of daily boating hours

**Figure 3:**
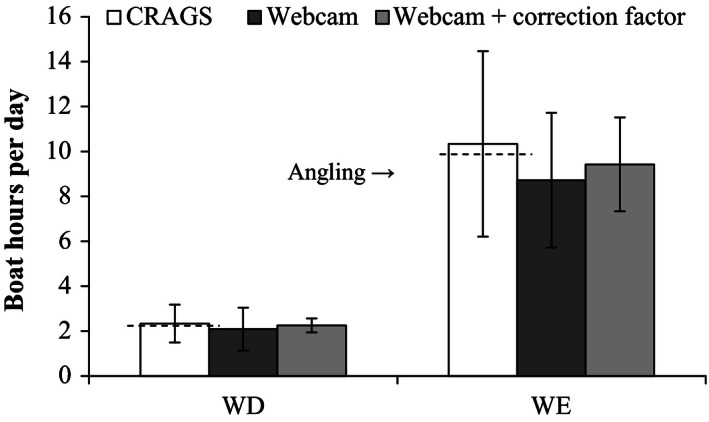
Comparison of estimates of average daily boating activity from 07:00 to 18:00 (± SE) on weekdays (WD;* n* = 12) and weekends (WE;* n* = 6) obtained using a CRAGS high‐resolution photo‐mosaic system, webcam and webcam with in situ data correction factor applied. Broken line on CRAGS indicates partition of angling (below line) and nonangling (above line) activity.

Excluding the dawn period, estimated daily hours of boating activity were similar between methods. CRAGS recorded 90 h of boating activity, 79.5 of which were angling compared with 77.3 h of angling recorded using the webcam and 83.5 h using the webcam with the correction factor (*Cf*). Four times more boating activity occurred on weekends (CRAGS *x̄* = 10.3, SE = 4.1; webcam *x̄* = 8.7, SE = 3) than weekdays (CRAGS *x̄* = 2.3, SE = 0.8; webcam *x̄* = 2.3, SE = 2.3; Fig. 3), with no effect or interaction between method and day type (two‐way repeated‐measures ANOVA: day type: *F*_1,16_) = 6.39, *P* < 0.05; method*day type *F*_1,16_ = 0.014, *P* > 0.05; method: *F*_1,16_ = 0.967, *P* > 0.05). HRPM from CRAGS revealed that types of use remained relatively consistent across the week, with recreational fishing boats accounting for 85% and other vessels 15% of weekday use. On weekends, recreational fishing boats accounted for 79%, charter fishing boats 3%, commercial fishing boats <1%, and other vessels 17% of use.

**Figure 4:**
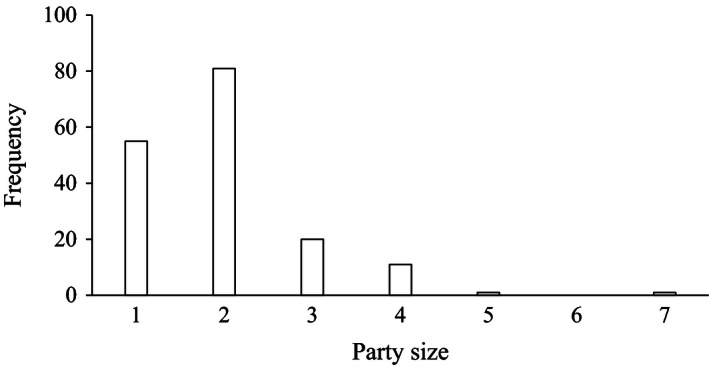
Frequency histogram of angler party sizes observed in high‐resolution photo‐mosaic (*n* = 169).

Overall CRAGS images were sharp enough to detect unobscured party members in 93% of cases. Mean party size (individuals per vessel) for recreational fishing boats was 1.97 (SE 0.07; Fig. 4), charter fishing boats 9.75 (SE 0.25), and other boats 3.46 (SE 0.79). Party size for “other boats” was likely to be an underestimate due to vessel structure obscuring people as these tended to be large vessels. Party size for the commercial boat could not be estimated due to these being in the 7% of poor image resolution data as well as the low number of samples for this activity type.

While initial costs were higher for the HRPM technology ($9000 for the CRAGS unit and $300 for stitching software vs. $900 webcam), labour costs to extract data from imagery was similar (~2 days FTE per week of data for both cameras).

## Discussion

For most time periods, the overall estimations of boating activity were similar between the HRPM produced by CRAGS and the Webcam systems, and statistical extrapolation of user hours provided the same information on fishing effort. While the webcam was able to take images more frequently than the CRAGS, the estimate of mean boat trip duration generated with the webcam (30.22 min, SE 3.37) was similar to the boat trip duration assumed using the CRAGS method (30 min). This suggests that the lower sample frequency is adequate for this application.. The sample frequency of CRAGS is ultimately limited by the time to shoot one panorama, 8 min in this case. The main advantage of the high‐resolution camera system is the capability to capture additional metrics, such as party size and user type (Pollock et al. 1994; VanDeValk et al. 2007) – which are unable to be collected with the low‐resolution web camera. The wider field of view of the high‐resolution camera also revealed that use of the OAR might extend past the survey area boundary used during all previous monitoring of the reef (NSW DPI, 2011; Keller et al. 2016). The boundary activities included deployment of commercial fishing traps, a boat deploying SCUBA divers at the site and slow‐moving boats, which appeared to be trolling for pelagic species. These boundary uses may impact on the OAR's ecological communities or associated recreational amenities.

The webcam uses variable frame rates to compensate for changing light environments allowing collection of data from the dawn period. This identified a previously unknown activity peak consistent outside of daytime hours (Smallwood et al. 2011), which we suspect was bait capture for pelagic game fishing. As with other recently established fisheries, use of the OAR has varied greatly with season and year since deployment (Lowry and Folpp 2014; Keller et al. 2016) and further monitoring on an interseasonal temporal scale is necessary to better understand patterns of use. The poor performance of the CRAGS method during the dawn period highlights the importance of site specific pilot studies when deploying new survey techniques. This technical limitation was not anticipated during our set up for what we assumed to be a daytime fishery. In future work, we would solve this by adjusting the ISO to perform better in low‐light conditions.

Robotic panoramic camera systems such as CRAGS do incur slight additional setup costs compared with traditional camera systems; however, they provide more detailed imagery across wider fields of view. The use of panoramic technology is hence most appropriate for monitoring sites where subjects are some distance from shore or cover a large area, while webcams or traditional camera trapping is better suited to smaller areas or “choke points” and there are a limited or easily discriminated number of user types such as boat ramps and jetties, which have known high levels of use by divers and fishers (Lynch et al. 2004; Smallwood et al. 2011). The time needed to analyze imagery may also benefit from the development of applicable computer‐based image‐differencing techniques (i.e., identifying changes between images) and automated image‐recognition systems (e.g., Coulter et al. 2011).

High‐definition panorama systems provide an opportunity to extend and enhance data collection opportunities, particularly for remote offshore systems, which are difficult to access. As there are plans to deploy two more offshore OARs in NSW over the next few years, being able to assess use at a fine scale will be valuable. In particular, understanding type of use may be important if competition between recreational (Lynch et al. 2004) or commercial users (Samples 1989) can occur at highly spatially constrained but well‐patronized sites.

## Data Accessibility

All data on use of the artificial reef used in this manuscript will be archived online at Dryad (http://datadryad.org).

## Conflict of Interest

None declared.
